# Assessing the meaningful change threshold of Quality of Life in Depression Scale using data from two phase 3 studies of esketamine nasal spray

**DOI:** 10.1186/s41687-022-00453-y

**Published:** 2022-07-10

**Authors:** Heather Rozjabek, Nan Li, Holger Hartmann, Dong Jing Fu, Carla Canuso, Carol Jamieson

**Affiliations:** 1 Janssen Global Services, LLC, Raritan, NJ USA; 2 Janssen Research and Development, LLC, Horsham, PA USA; 3 Health Economics and Market Access Research, Janssen-Cilag, Neuss, North Rhine-Westphalia Germany; 4 Janssen Research and Development, LLC, Titusville, NJ USA; 5 Janssen Research and Development, LLC, Milpitas, CA USA

**Keywords:** Quality of Life in Depression, Major depressive disorder, Meaningful change threshold, Anchor-based approach

## Abstract

**Background:**

Major depressive disorder (MDD) directly impacts patients’ lives including symptoms, functioning and health-related quality-of-life (HRQoL). Patient-reported outcomes can capture these impacts, however interpretation of clinical meaningfulness of these measurements are often not readily available. Meaningful change thresholds (MCTs) can be derived for clinical outcome assessments to quantify the change in symptoms that is meaningful to the patient following pharmacologic treatment or other intervention**s**. The objective of this analysis was to determine the within-patient MCT of the self-reported Quality-of-Life in Depression Scale (QLDS) among patients with MDD and active suicidal ideation with intent (MDSI) using an anchor-based approach.

**Methods:**

Data from 2 randomized phase-3 trials of esketamine nasal spray (ASPIRE I and ASPIRE II) were analyzed. The Montgomery–Åsberg Depression Rating Scale (MADRS) was the primary anchor with three different severity criteria. Other anchor variables utilized were Clinical Global Impression of Severity of Suicidality-revised version, Clinical Global Impression of Imminent Suicide Risk, and EuroQol Visual Analog Scale [EQ-VAS]. Spearman correlation coefficients between the change in QLDS and anchor variables were calculated. The mean change in QLDS score at Day 25 from baseline was calculated based on the categorical change in the anchor. Coefficient yield from linear regression of the mean changes in EQ-VAS and QLDS, and distribution-based approach with ½ SD of change in QLDS were considered.

**Results:**

In ASPIRE I, mean (SD) improvement in QLDS score among patients with one category improvement in MADRS from baseline to Day 25 was − 8.22 (8.87), − 8.30 (9.01), and − 8.20 (8.92) using severity criteria #1, #2, and #3, respectively. Patients who achieved a 7-point improvement (MCT) in EQ-VAS yielded a mean − 9.69-point improvement in QLDS at Day 25. The ½ SD of change in QLDS was 5.63. Similar results were obtained for ASPIRE II. The MCTs identified using multiple anchors across both trials ranged from − 11.4 to − 6.7 and had an overall mean of − 7.90 (ASPIRE I) and − 7.92 (ASPIRE II). Thus, an 8-point change was recommended as the MCT for QLDS.

**Conclusion:**

The recommended MCT will help quantify within-person changes in HRQoL using patient-reported QLDS and determine meaningful treatment benefit in an MDD patient population with acute suicidal ideation or behavior.

*Trial registration*: Name of the registry: ClinicalTrials.gov. Trial registration number: ASPIRE I (NCT03039192), ASPIRE II (NCT03097133). Date of registration: February 01, 2017; March 31, 2017. URL of trial registry record: https://clinicaltrials.gov/ct2/show/NCT03039192; https://clinicaltrials.gov/ct2/show/NCT03097133.

**Supplementary Information:**

The online version contains supplementary material available at 10.1186/s41687-022-00453-y.

## Introduction

Major depressive disorder (MDD) is a common mental health condition affecting over 163 million people worldwide [1] and over 21 million people in the United States [2]. The major symptoms of MDD include low mood, lack of energy, insomnia, sadness, and inability to enjoy life [3]. MDD also affects normal functioning as it reduces health-related quality-of-life (HRQoL) [4] and productivity at work [5]. MDD is also associated with suicide ideation [2]; and the prevalence of suicide attempts in patients with a lifetime MDD episode in the United States is reported to be 13.6% [6].

Despite availability of multiple treatments, first-line agents fail to adequately treat MDD in many patients [7], which raises the question whether existing methods of evaluating depression and its treatment in clinical trials are sufficiently comprehensive. Gathering patients’ perspectives provide valuable insights on potential depression symptoms, day to day problems, health-related quality of life and treatment satisfaction and is a promising approach to address currently unmet needs [8]. Optimizing patient-reported outcomes (PRO) in clinical trials and improving their interpretation is therefore gaining momentum [9]. Kamenov et al. [10] analyzed 247 interventional studies for depression and reported that 80% of the areas covered by the employed PROs and clinician-rated tools represented clinical symptomatology, while other areas of functioning such as daily routine, work activities, and social participation were insufficiently or hardly covered. Since patients seem to prioritize functional outcomes over symptomatic outcomes [11], there is increasing emphasis on incorporating functional outcomes into clinical trials [12].

Patient-reported outcome measures can effectively capture the impact of a disease on the patient’s daily life, including symptoms, functioning, and overall HRQoL [13–15]. The Quality of Life in Depression Scale (QLDS) is a disease-specific patient-reported questionnaire designed to assess the impact of depression on the HRQoL of patients [16]. The QLDS has been shown to have high reliability and construct validity [17], has been translated into multiple languages [18], and has been used in clinical trials and observational studies for the past 2 decades [19–27]. However, it is important to understand whether the results from a PRO are meaningful from the patients’ perspective. Critical Path Institute’s PRO Consortium and the Consensus Panel for Outcomes Measurement and Psychometrics: Advancing the Scientific Standards (COMPASS) recommends the use of the term meaningful change threshold (MCT), which describes the threshold of change at which the change becomes meaningful to the patient [28, 29]. According to these guidelines, the preferred method for deriving MCTs in a clinical trial and regulatory setting is to use anchor-based approaches. The approach is used to estimate mean change in a PRO measure in patients who reported a magnitude of change consistent with a clinically relevant change in the global impression of disease severity or health measure, with supportive cumulative distribution and probability density function curves [14, 15, 30, 31].

Establishing MCTs for clinical outcome assessments are essential for interpreting treatment effects; therefore, the objective of this analysis was to determine the within-person MCT for QLDS among patients with MDD who have active suicidal ideation with intent (MDSI), using data from two phase 3 studies of esketamine nasal spray (ESK).

## Methods

### Study selection

Data for this secondary analysis were obtained from two identically designed, randomized, double-blind, placebo-controlled, multicenter, phase 3 studies (ASPIRE I [NCT03039192] and ASPIRE II [NCT03097133]) that evaluated efficacy and safety of ESK + standard of care (SOC) versus placebo + SOC. Methods and primary data of these studies have been reported earlier [32, 33].

In brief, these two studies included men and women (aged 18–64 years) who were diagnosed with MDD (as per Diagnostic and Statistical Manual of Mental Disorders, 5th Edition) [34] without psychotic features as confirmed by the Mini International Neuropsychiatric Interview [35]; had current suicidal ideation with intent within 24 h prior to randomization, as confirmed by responding ‘Yes’ to the questions “Think about suicide?” and “Intend to act on thoughts of killing yourself?”; were in need of acute psychiatric hospitalization due to imminent risk of suicide; and had a Montgomery–Åsberg Depression Rating Scale (MADRS) [36] total score > 28 pre-dose on day 1.

The patients went through an initial screening phase conducted within 48 h prior to Day 1 dose, followed by a 4-week double-blind treatment phase wherein patients were randomized (1:1) to receive ESK (84 mg) + SOC or placebo + SOC twice weekly followed by a 9-week follow-up phase. In both ASPIRE I and II, significant improvement in MADRS total score from Baseline at 24 h (primary endpoint) was observed in patients receiving ESK + SOC versus placebo + SOC (least-squares mean difference [SE]: ASPIRE I, − 3.8 [1.39], 95% CI: − 6.56, − 1.09; *p* = 0.006; ASPIRE II, − 3.9 [1.39], 95% CI: − 6.60, − 1.11; *p* = 0.006) [37, 38].

### Study measures

#### QLDS

The QLDS is a disease-specific patient-reported outcome designed to assess HRQoL in patients with MDD [16, 17]. The instrument has a recall period of "at the moment," contains 34-items with “true”/ “not true” response options and takes approximately 5–10 min to complete. The score range is from 0 (good quality of life) to 34 (very poor quality of life) [17]. It has been shown to have acceptable psychometric properties and sensitivity to change [17, 18]. The QLDS was self-administered in the participant’s native language and in electronic format using a computer tablet in both ASPIRE trials.

#### MADRS

The MADRS is a 10-item clinician-rated scale designed to measure depression severity [36, 39], with each item scored from 0 (item not present or normal) to 6 (severe or continuous presence of symptoms) for a total possible score range of 0 to 60. The MADRS evaluates apparent sadness, reported sadness, inner tension, sleep, appetite, concentration, lassitude, interest level, pessimistic thoughts, and suicidal thoughts. Since there is no consensus on how to categorize MADRS for different severity levels of depression, three sets of cut-offs were adopted in this analysis **(**Table 1) [40–42].

**Table 1:** MADRS severity criteria

Criteria #1 and cut-off values [42]	Criteria #2 and cut-off values [41]	Criteria #3 and cut-off values [40]
No depression (0–12)	No depression (0–6)	No depression (0–12)
Slight depression (13–21)	Slight depression (7–19)	Slight depression (13–17)
Moderate depression (22–28)	Moderate depression (20–34)	Moderate depression (18–34)
Severe depression (> 28–60)	Severe depression (> 34–60)	Severe depression (> 34–60)

*MADRS* Montgomery–Åsberg Depression Rating Scale

#### CGI-SS-r

The CGI-SS-r is included in Module 7 of the Suicide Ideation and Behavior Assessment Tool (SIBAT) [43]. The SIBAT is a computerized assessment tool with five patient-reported and three clinician-reported modules that has been designed to systematically and comprehensively capture suicidal ideation and behavior (SIB), and measure rapid changes in SIB. After collection of relevant background information using Modules 1–5, clinicians conduct a semi-structured interview (Module 6) and finalize ratings for four outcome measures in Module 7, followed by documentation in Module 8 [43]. The CGI-SS-r in Module 7 summarizes the clinician’s overall impression of severity of suicidality on a 7-point scale (0-normal, 1-questionably, 2-mildly, 3-moderately, 4-markedly, 5-severely, and 6-most extremely suicidal) based on the totality of information available to the clinician, including information from the completed modules of the SIBAT. The category ratings in the CGI-SS-r are directly interpretable as different levels of suicidality and a 1-point change in a CGI scale is consistent with a clinically observable change [44, 45].

#### CGI-SR-I

The CGI-SR-I (included in Module 7 of the SIBAT) summarizes the clinician’s best assessment of the likelihood that a patient will attempt suicide in the next 7 days on a 7-point scale (0-no imminent suicide risk to 6-extreme imminent suicide risk) [43].

#### EQ-VAS

The EQ-VAS is a part of the European Quality of Life Group-5 Dimension 5-Level questionnaire, a measure for HRQoL designed for self-completion by the respondent [46]. Patients self-rated their overall health status from 0 (worst health) to 100 (best health). Changes in EQ-VAS on the order of 7 to 10 were recognized as a threshold for meaningful change for an individual patient [47].

### Assessment of MCT

Various methods, including anchor-based and distribution-based approaches, can be used to determine a meaningful change threshold. The anchor-based approach compares the change in the measure under evaluation to another measure of change, considered an anchor or external criterion, while the distribution-based approach compares the change in the measure under evaluation to a measure of variability (e.g. standard error of measurement or standard deviation) [48]. While both methods can be used to measure meaningful change, anchor-based methods are preferred to derive an MCT and distribution-based thresholds can be supportive [30]. The choice of an MCT is often through a multifaceted triangulation of analytic approaches which may include feedback from experts, including clinicians and patients, which cannot be prescribed in an analysis plan [49].

Full efficacy analysis datasets were utilized for the MCT analysis, which had 224 patients for ASPIRE I and 227 patients for the ASPIRE II study. Treatment groups for individual studies were combined for the current analysis. The MCT was assessed using primarily an anchor-based approach with multiple anchors. The MADRS was used as the primary anchor. Three variable sets of score criteria to determine severity levels for depression on the MADRS are reported in the literature; therefore, all 3 scoring criteria were evaluated in this analysis to evaluate consistency. The three severity criteria are as follows: severity criteria #1 [42]– no depression (score 0–12), slight depression (score 13–21), moderate depression (score 22–28), and severe depression (score > 28–60); severity criteria #2 [41]– no depression (score 0–6), mild depression (score 7–19), moderate depression (score 20–34), and severe depression (score > 34–60); and severity criteria #3 [40]– no depression (score 0–12), mild depression (score 13–17), moderate depression (score 18–34), and severe depression (score > 34–60) **(**Table 1). Other potential anchor variables were the Clinical Global Impression of Severity of Suicidality-Revised (CGI-SS-r) [43], the Clinical Global Impression of Imminent Suicide Risk (CGI-SR-I) [43], and EuroQol Visual Analog Scale (EQ-VAS) [46] were also explored. Distribution based MCT was calculated using 1/2 standard deviation (SD) of change in QLDS score.

### Statistical analysis

Descriptive statistics of the mean QLDS total scores and mean change from Baseline to Day 25 (end point of the double-blind treatment phase) are presented. Spearman correlation coefficients between the change in QLDS score and the change in anchor variables from Baseline to Day 25 were calculated to confirm the relevance of the anchor. A correlation coefficient of ≥ 0.40 was needed for the anchor to be used in this analysis [50]. Change of QLDS score between Baseline and Day 25 was calculated in patients within change groups defined by the anchor variable. Mean (SD) within-patient change from Baseline, along with the median, 95% confidence intervals, standardized response mean, and standardized effect size were calculated for each of the categories of change in the anchor variable from Baseline. Coefficients yielded from linear regression of EQ-VAS and QLDS scores were used to provide additional data to evaluate the MCT of QLDS. Cumulative distribution function (CDF) curves were also generated of the change in QLDS score from Baseline to Day 25 stratified by MADRS change category at Day 25. The CDF curves provide a graphic representation of the full distribution of the QLDS change scores between MADRS change anchor categories, which provide additional support for the selection of the anchor category where the change in QLDS is observed.

## Results

### ASPIRE I results

The mean (SD) age of the patients at Baseline was 39.3 (12.9) years and most of the patients were women (61.6%). Majority of the patients (88.8%) were considered by clinicians to be moderately to extremely suicidal while 84.8% of the patients were considered to have moderate to extreme imminent risk for suicide within the next seven days (Table 2). The mean (SD) MADRS score at Baseline was 41.1 (6.07), which improved to 17.9 (12.09) at Day 25 for a mean change of -23.2 (13.03). The mean (SD) QLDS score decreased from 27.24 (6.40) at Baseline to 14.79 (11.33) at Day 25; mean (SD) change of − 12.46 (11.26).

**Table 2:** Summary of demographics and anchor variables at Baseline

Parameter	ASPIRE I (n = 224)	ASPIRE II (n = 227)
*Age, years*		
Mean (SD)	39.3 (12.91)	40.8 (13.07)
Range	18–64	18–64
*Sex, n *(%)		
Women	138 (61.6)	136 (59.9)
Men	86 (38.4)	91 (40.1)
*QLDS*		
Mean (SD)	27.24 (6.40)	26.77 (5.64)
Range	0–34	5–34
*MADRS score*		
Mean (SD)	41.1 (6.07)	39.7 (5.48)
Range	29–58	29–54
*CGI-SS-r, n* (%)		
Normal, not at all suicidal	0	0
Questionably suicidal	8 (3.6)	4 (1.8)
Mildly suicidal	17 (7.6)	16 (7.0)
Moderately suicidal	57 (25.6)	68 (30.0)
Markedly suicidal	80 (35.9)	90 (39.6)
Severely suicidal	56 (25.1)	45 (19.8)
Among the most extremely suicidal patients	5 (2.2)	4 (1.8)
*CGI-SR-I, n* (%)		
No imminent suicide risk	5 (2.2)	3 (1.3)
Minimal imminent suicide risk	12 (5.4)	8 (3.5)
Mild imminent suicide risk	17 (7.6)	18 (7.9)
Moderate imminent suicide risk	57 (25.6)	63 (27.8)
Marked imminent suicide risk	75 (33.6)	80 (35.2)
Severely imminent suicide risk	53 (23.8)	49 (21.6)
Extreme imminent suicide risk	4 (1.8)	6 (2.6)
*EQ-VAS*		
Mean (SD)	42.0 (24.44)	39.5 (22.62)
Range	0–100	0–95

*CGI-SR-I* Clinical Global Impression of Imminent Suicide Risk, *CGI-SS-r* Clinical Global Impression of Severity of Suicidality-Revised, *EQ-VAS* EuroQol Visual Analog Scale, *MADRS* Montgomery–Åsberg Depression Rating Scale, *QLDS* Quality of Life Depression Scale, *SD* Standard Deviation

**Fig. 1:**
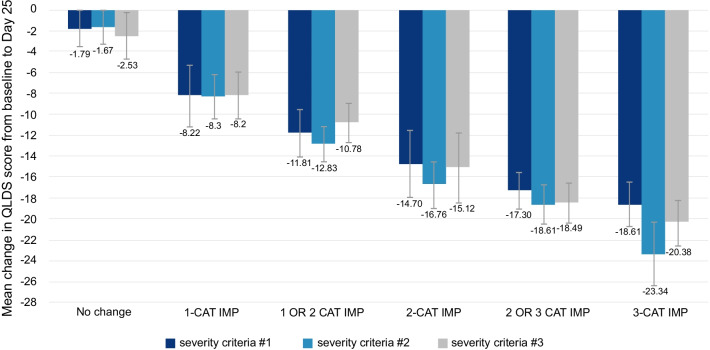
ASPIRE I–mean change in QLDS score from baseline to Day 25 using MADRS as an anchor. Values are represented as mean ± 95% CI. MADRS was categorized as: severity criteria #1—no depression (score 0–12), slight depression (score 13–21), moderate depression (score 22–28), and severe depression (score > 28–60); severity criteria #2—no depression (score 0–6), mild depression (score 7–19), moderate depression (score 20–34), and severe depression (score > 34–60); and severity criteria #3—no depression (score 0–12), mild depression (score 13–17), moderate depression (score 18–34), and severe depression (score > 34–60). *CAT* Category, *CI* Confidence Interval, *IMP* Improvement, *MADRS* Montgomery–Åsberg Depression Rating Scale, *QLDS* Quality of Life in Depression Scale

The correlation between change in QLDS score and change in MADRS total score at Day 25 was 0.66 (*p* < 0.0001). The mean (SD) improvement in QLDS score from Baseline to Day 25 among patients who improved one, two, and three MADRS severity categories (as previously defined) was slightly different using alternative MADRS criteria: − 8.22 (8.87), − 14.70 (10.85), and − 18.61 (10.15), respectively, using severity criteria #1; − 8.30 (9.01), − 16.76 (10.28), and − 23.34 (8.42), respectively, using severity criteria #2; and − 8.20 (8.92), − 15.12 (10.80), and − 20.38 (9.41), respectively, using severity criteria #3 (Fig. 1; Table S1 in Additional file 1).

The correlation between change in QLDS score and CGI-SS-r score at Day 25 was 0.43 (*p* < 0.0001). The mean (SD) improvement in QLDS score from Baseline to Day 25 among patients who improved one, two, and three points in CGI-SS-r was − 8.20 (9.13), − 9.74 (10.54), and − 12.33 (11.75), respectively. The correlation between change in QLDS score and CGI-SR-I score at Day 25 was 0.44 (*p* < 0.0001). The mean (SD) improvement in QLDS score from Baseline to Day 25 among patients who improved one, two, and three points in CGI-SR-I was − 6.06 (8.33), − 10.84 (9.81), and − 12.55 (11.20), respectively.

The mean (SD) Baseline EQ-VAS score was 42.0 (24.44), which improved to 62.7 (21.76) at Day 25; mean change of 20.7 (25.15). The correlation between change in QLDS score and change in EQ-VAS score (0–100) between Baseline and Day 25 was − 0.53 (*p* < 0.0001). Based on the linear regression of QLDS on EQ-VAS (β_0_ = -8.00, *p* < 0.0001; β_1_ =  − 0.24, *p* < 0.0001), a 7-point improvement in EQ-VAS score—which is the MCT for EQ-VAS—led to a − 9.69-point improvement in QLDS score. The MCT for QLDS using the distribution-based approach was 5.63 with 1/2 SD of change in QLDS score between Baseline and Day 25.

### ASPIRE II results

The mean (SD) age of the patients at Baseline was 40.8 (13.1) years and most of the patients were women (59.9%). Majority of the patients (91.2%) were considered by clinicians to be moderately to extremely suicidal while 87.2% of the patients were considered to have moderate to extreme imminent risk for suicide within the next seven days (Table 2). The mean (SD) MADRS score at Baseline was 39.7 (5.48), which improved to 17.3 (11.73) at Day 25 for a mean change of -22.33 (12.65). The mean (SD) QLDS score decreased from 26.77 (5.64) at Baseline to 14.07 (11.02) at Day 25; mean (SD) change of − 12.66 (11.20).

**Fig. 2:**
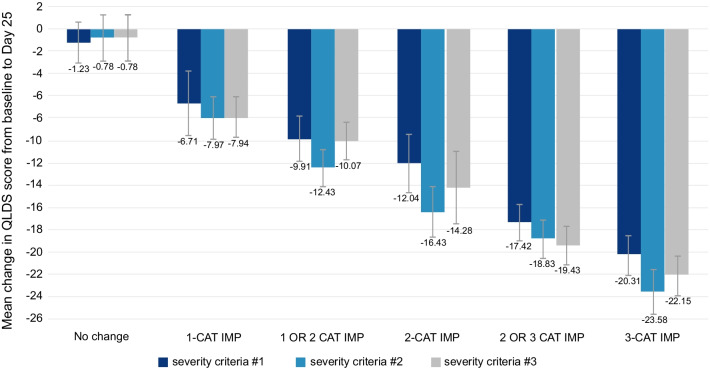
ASPIRE II–mean change in QLDS score from baseline to Day 25 using MADRS as an anchor. Values are represented as mean ± 95% CI. MADRS was categorized as: severity criteria #1—no depression (score 0–12), slight depression (score 13–21), moderate depression (score 22–28), and severe depression (score > 28–60); severity criteria #2—no depression (score 0–6), mild depression (score 7–19), moderate depression (score 20–34), and severe depression (score > 34–60); and severity criteria #3—no depression (score 0–12), mild depression (score 13–17), moderate depression (score 18–34), and severe depression (score > 34–60). *CAT* Category, *CI* Confidence Interval, *IMP* Improvement, *MADRS* Montgomery–Åsberg Depression Rating Scale, *QLDS* Quality of Life in Depression Scale

The correlation between the change in QLDS score and change in MADRS category at Day 25 was 0.70 (*p* < 0.0001). The mean (SD) improvement in QLDS score from Baseline to Day 25 among patients who improved one, two, and three categories was slightly different using alternative MADRS criteria: − 6.71 (8.33), − 12.04 (9.26), and − 20.31 (8.92), respectively, using severity criteria #1; − 7.97 (8.10), − 16.43 (10.31), and − 23.58 (6.32), respectively, using severity criteria #2; and − 7.94 (8.21), − 14.28 (10.24), and − 22.15 (7.67), respectively, using severity criteria #3 (Fig. 2; Table S2 in Additional file 1).

The correlation between change in QLDS score and CGI-SS-r at Day 25 was 0.43 (*p* < 0.0001). The mean (SD) improvement in QLDS score from Baseline to Day 25 among patients who improved one, two and three categories in CGI-SS-r was − 4.81 (7.27), − 11.28 (10.13), − 14.65 (11.14), respectively. The correlation between change in QLDS score and CGI-SR-I score at Day 25 was 0.41 (*p* < 0.0001). The mean (SD) improvement in QLDS score from Baseline to Day 25 among patients who improved one, two, and three categories in CGI-SR-I was − 10.27 (11.18), − 9.08 (10.23), and − 13.57 (10.26), respectively.

The mean (SD) Baseline EQ-VAS score was 39.5 (22.62), which improved to 62.12 (23.18) at Day 25; mean change of 22.7 (26.73). The correlation between change in QLDS score and change in EQ-VAS score between Baseline and Day 25 was − 0.62 (*p* < 0.0001). A 7-point improvement in EQ-VAS score led to a − 9.66-point improvement in QLDS score in the regression model (β_0_ =  − 7.84, *p* < 0.0001; β_1_ =  − 0.26, *p* < 0.0001). The MCT of QLDS using the distribution-based approach was − 5.60 with 1/2 SD of change in QLDS score between Baseline and Day 25.

### MCT for QLDS

**Fig. 3:**
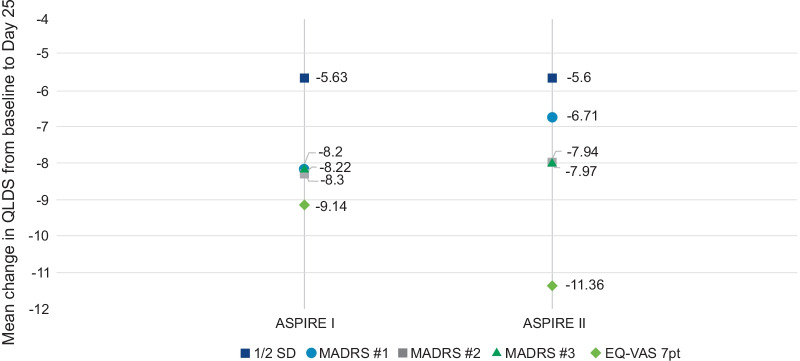
Meaningful change thresholds of QLDS using anchor- and distribution-based approach. Values represent MCT for various categories or points of improvement in QLDS score from Baseline to Day 25. For all MADRS severity criteria—one-category improvement and for EQ-VAS—7-point improvement. *EQ-VAS* EuroQol Visual Analog Scale, *MADRS* Montgomery–Åsberg Depression Rating Scale, *MCT* meaningful change threshold; *QLDS* Quality of Life in Depression Scale, *SD* Standard Deviation

**Fig. 4:**
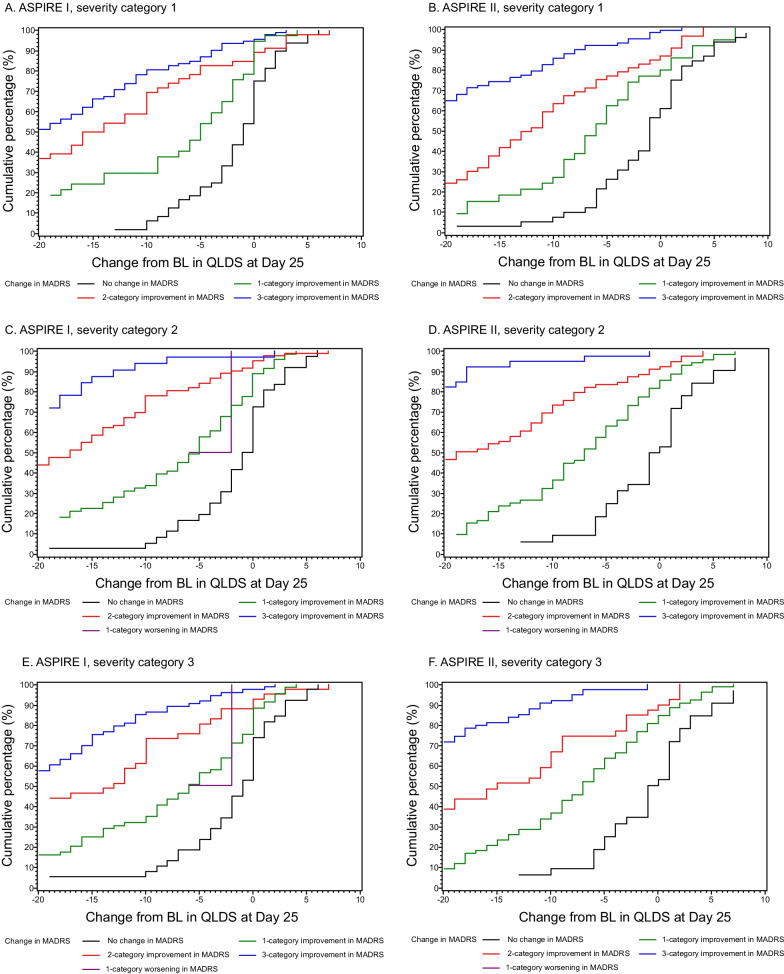
Cumulative distribution function of change from baseline to Day 25 in QLDS score stratified by MADRS change category at Day 25. **A**, **B**: Severity Criteria #1; **C**, **D**: Severity criteria #2; **E**, **F**: Severity Criteria #3 (see Table 1 for details). *BL* Baseline, *IMP* Improvement, *MADRS* Montgomery–Åsberg Depression Rating Scale, *QLDS* Quality of Life in Depression Scale

The MADRS and the EQ-VAS anchors had the strongest correlations with the change in QLDS score at Day 25 compared to the CGI-SS-r and CGI-SR-I. Therefore, the MADRS and EQ-VAS anchors were considered more predominantly in the determination of the MCT. The MADRS was the primary anchor variable with the EQ-VAS, CGI-SS-r and CGI-SR-I used as supporting anchor measures. The results obtained using the supporting anchors and the distribution results were consistent with the MADRS anchor-based results. Considering the MCTs for the QLDS score identified using the MADRS and EQ-VAS, the mean improvement in QLDS score was − 7.90 for the ASPIRE I study and − 7.92 for the ASPIRE II study. The MCTs obtained from the MADRS and EQ-VAS anchor-based analysis and the distribution-based approach for both ASPIRE I and ASPIRE II are summarized in Fig. 3. In both trials, a separation was observed on CDF curves between different MADRS change category groups with all three MADRS severity criteria **(**Fig. 4).

## Discussion

This study aimed to determine the within-person MCT for QLDS among patients with MDD and acute suicidal ideation or behavior using data from two separate phase-3 trials of esketamine nasal spray. Using multiple anchors and distribution-based approaches, our findings suggest that within-patient MCT of QLDS among patients with MDD and acute suicidal ideation or behavior ranged from − 11.4 to − 6.7. The median of MCTs obtained using various approaches were − 8.09 (mean =  − 7.9). Therefore, we recommend using an 8-point change as an MCT for the QLDS.

Evidence for an MCT of − 8 points on the QLDS was supported using multiple anchors, including both patient-reported and clinician-reported outcomes measures, and distribution-based approaches. Although the MCT can be determined using either an anchor-based or distribution-based approach [48], we chose to utilize both methods for validation. Results from other anchor-based analyses such as the CGI-SS-r and CGI-SR-I provided additional information for the selection of the most appropriate MCT. For both the CGI-SR-I and the CGI-SS-r in ASPIRE II, the number of patients who improved only one category was small (26 and 21 respectively), which may have contributed to the variability in the results. The median QLDS score change for a one category change in CGI-SR-I and CGI-SS-r was − 9.0 and − 5.0, respectively. These findings are further supported by the separation of CDF curves between the MADRS change categories. CDF curves demonstrated a clear separation in the distribution of QLDS score between no change and a 1, 2 or 3-category improvement in MADRS scores. Although the distribution-based methods have been previously utilized in MCT analyses, these methods are sensitive to the homogeneity of the distribution and can result in MCTs which may be too small of a change to be considered meaningful. Use of both approaches in this analysis allowed for a range of MCT values to help interpret meaningful within-patient change. Studies have reported the inadequate coverage of outcomes integral from the patient’s and clinician’s point of view in research, as symptomatic outcomes are often the only outcome of interest in clinical trials [10, 51]. Lack of quantitative measures in patient-reported outcomes instruments to distinguish between treatment responders and non-responders, as well as inadequate implementation of such instruments, could be a reason for the aforementioned bias towards symptomatic outcomes [51]. However, the concept of MCT has been increasingly utilized to evaluate pharmacological interventions across therapy areas [52, 53]. MCTs have also been developed for many depression rating scales such as the MADRS, 9-item Patient Health Questionnaire, Beck Depression Inventory and others [54–57]. Our study adds a quantitative aspect to the QLDS and will assist researchers and clinicians in assessing the benefit of a therapy. The reported MCT values are generalizable to the study population in which they were derived, which is adult patients with MDD requiring acute psychiatric hospitalization due to an imminent suicide risk.

### Limitations

A potential limitation of this study is that a patient-reported global impression of change or severity in depression was not available for use as an anchor for deriving the MCT. Instead, MADRS and other clinician global impression anchors were used to assess symptoms and behavior of the patient. The EQ-VAS, which is patient-reported, can assess a patient’s overall assessment of their health; however, it would still be important to confirm the MCT in future studies where a depression specific patient global impression anchor is available. Although we obtained the MCT using data from two phase-3 trials among patients with MDSI, further research should be conducted to confirm these results in a similar MDSI population and evaluate an MCT value on the QLDS within a general MDD population.

## Conclusion

Analysis of data from two double-blind, randomized, phase-3 trials among patients with MDD and acute suicidal ideation or behavior resulted in an MCT of 8-point improvement for the QLDS. This information is potentially useful to researchers and clinicians in interpreting changes in the HRQoL as measured by the QLDS among those with MDD and acute suicidal ideation or behavior.

## Supplementary Information

**Additional file 1.** **Table 1.** Results – MADRS as anchor in ASPIRE I. **Table 2.** Results – MADRS as anchor in ASPIRE II.

## Data Availability

The data sharing policy of Janssen Pharmaceutical Companies of Johnson & Johnson is available at https://www.janssen.com/clinicaltrials/ transparency. As noted on this site, requests for access to the study data can be submitted through Yale Open Data Access (YODA) Project site at http://yoda.yale.edu.
