# Supplementary material for: Assessing the meaningful change threshold of Quality of Life in Depression Scale using data from two phase 3 studies of esketamine nasal spray
Source: J Patient Rep Outcomes. 2022 Jul 10;6:74. doi: 10.1186/s41687-022-00453-y (PMC9273809; doi:10.1186/s41687-022-00453-y)
Supplement: Supplementary file 1 — Additional file 1. Table 1. Results – MADRS as anchor in ASPIRE I. Table 2. Results – MADRS as anchor in ASPIRE II. [file 41687_2022_453_MOESM1_ESM.docx]

**Table 1: Results – MADRS as anchor in ASPIRE I**

| **Change in QLDS** | **2 or 3 -CAT IMP** | **3 -CAT IMP.** | **1 or 2 - CAT IMP** | **2- CAT IMP** | **1- CAT IMP** | **No change** |
| --- | --- | --- | --- | --- | --- | --- |
| **MADRS as anchor ^a^ (severity criteria #1)** | | | | | | |
| **n** | 138 | 92 | 83 | 46 | 37 | 48 |
| **Mean Baseline** | 26.21 | 25.55 | 28.18 | 27.52 | 29.00 | 28.88 |
| **95% CI** | 25.01, 27.41 | 23.99, 27.12 | 27.00, 29.36 | 25.73, 29.31 | 27.51, 30.49 | 27.51, |
| **SD** | 7.13 | 7.57 | 5.41 | 6.03 | 4.47 | 4.68 |
| **Mean change** | -17.30 | -18.61 | -11.81 | -14.70 | -8.22 | -1.79 |
| **95% CI** | -19.07, -15.54 | -20.71, -16.51 | -14.09, -9.52 | -17.92, -11.47 | -11.18, -5.26 | -3.00, -0.59 |
| **SD** | 10.51 | 10.15 | 10.47 | 10.85 | 8.87 | 4.15 |
| **Median** | -18.00 | -20.00 | -10.00 | -15.00 | -5.00 | -1.00 |
| **SRM** | -1.65 | -1.83 | -1.13 | -1.35 | -0.93 | -0.43 |
| **SES** | -2.43 | -2.46 | -2.18 | -2.44 | -1.84 | -0.38 |
| **MADRS as anchor ^b^ (severity criteria #2)** | | | | | | |
| **n** | 114 | 32 | 153 | 82 | 71 | 36 |
| **Mean Baseline** | 26.66 | 27.63 | 26.95 | 26.28 | 27.72 | 27.94 |
| **95% CI** | 25.43, 2.89 | 25.64, 29.61 | 25.83, 28.06 | 24.74, 27.82 | 26.08, 29.35 | 26.46, 29.43 |
| **SD** | 6.64 | 5.49 | 6.99 | 7.03 | 6.91 | 4.40 |
| **Mean change** | -18.61 | -23.34 | -12.83 | -16.76 | -8.30 | -1.67 |
| **95% CI** | -16.71, -20.50 | -26.38, -20.31 | -14.52, -11.14 | -19.02, -14.50 | -10.43, -6.16 | -3.30, -0.03 |
| **SD** | 10.20 | 8.42 | 10.57 | 10.28 | 9.01 | 4.83 |
| **Median** | -20 | -24.5 | -12 | -10 | -5 | -0.5 |
| **SRM** | -1.82 | -2.77 | -1.21 | -1.63 | -0.92 | -0.34 |
| **SES** | -2.80 | -4.25 | -1.84 | -2.38 | -1.20 | -0.38 |
| **MADRS as anchor ^c^ (severity criteria #3)** | | | | | | |
| **n** | 114 | 73 | 110 | 41 | 69 | 38 |
| **Mean Baseline** | 26.14 | 26.88 | 27.16 | 24.83 | 28.55 | 27.97 |
| **95% CI** | 24.81, 27.47 | 25.44, 28.31 | 25.81, 28.52 | 22.10, 27.56 | 27.16, 29.94 | 26.54, 29.41 |
| **SD** | 7.18 | 6.14 | 10.51 | 8.66 | 5.78 | 4.36 |
| **Mean change** | -18.49 | -20.38 | -10.78 | -15.12 | -8.20 | -2.53 |
| **95% CI** | -20.39, -16.60 | -22.58, -18.19 | -12.71, -8.86 | -18.53, -11.71 | -10.35, -6.06 | -4.73, -0.32 |
| **SD** | 10.21 | 9.41 | 10.19 | 10.80 | 8.92 | 6.70 |
| **Median** | -20.0 | -21.0 | -9.0 | -13.0 | -6.0 | -1.0 |
| **SRM** | -1.81 | -2.17 | -1.06 | -1.40 | -0.92 | -0.38 |
| **SES** | -2.58 | -3.32 | -1.03 | -1.75 | -1.42 | -0.58 |

^a^ MADRS is grouped as no depression (scores 0 to 12), slight depression (scores 13 to 21), moderate depression (scores 22 to 28) and severe depression (scores >28 to 60); ^b^ MADRS is grouped no depression (scores 0 to 6), mild (scores 7 to 19), moderate (scores 20 to 34) and severe (scores >34 to 60); ^c^ MADRS is grouped as no depression (scores 0 to 12), mild (scores 13 to 17), moderate (scores 18 to 34) and severe (scores >34 to 60); *CAT* category, *CI* confidence interval, *IMP* improvement, *MADRS* Montgomery–Åsberg Depression Rating Scale, *SD* standard deviation, *SRM* standardized response mean, *SES* standardized effect size

**Table 2: Results – MADRS as anchor in ASPIRE II**

| **Change in QLDS** | **2 or 3 - CAT IMP** | **3 - CAT IMP.** | **1 or 2 - CAT IMP** | **2- CAT IMP** | **1- CAT IMP** | **No change** |
| --- | --- | --- | --- | --- | --- | --- |
| **MADRS as anchor ^a^ (severity criteria #1)** | | | | | | |
| **n** | 146 | 95 | 85 | 51 | 34 | 43 |
| **Mean Baseline** | 26.70 | 26.59 | 26.51 | 26.90 | 25.91 | 27.67 |
| **95% CI** | 25.75, 27.64 | 25.40, 27.78 | 25.25, 27.77 | 25.29, 28.51 | 23.80, 28.02 | 26.20, 29.15 |
| **SD** | 5.77 | 5.83 | 5.84 | 5.72 | 6.04 | 4.78 |
| **Mean change** | -17.42 | -20.31 | -9.91 | -12.04 | -6.71 | -1.23 |
| **95% CI** | -19.03, -15.81 | -22.12, -18.49 | -11.90, -7.91 | -14.64, -9.43 | -9.61, -3.80 | -3.02, 0.55 |
| **SD** | 9.84 | 8.92 | 9.23 | 9.26 | 8.33 | 5.79 |
| **Median** | -19.5 | -23.0 | -9.0 | -12.0 | -6.0 | -1.00 |
| **SRM** | -1.77 | -2.27 | -1.09 | -1.30 | -0.85 | -0.21 |
| **SES** | -3.01 | -3.47 | -1.72 | -2.10 | -1.17 | -0.26 |
| **MADRS as anchor ^a^ (severity criteria #2)** | | | | | | |
| **n** | 119 | 40 | 150 | 79 | 71 | 32 |
| **Mean Baseline** | 27.54 | 27.28 | 26.69 | 27.67 | 25.61 | 26.66 |
| **95% CI** | 26.66, 28.41 | 26.05, 28.50 | 25.75, 27.64 | 26.49, 28.85 | 24.11, 27.10 | 24.31, 29.01 |
| **SD** | 4.82 | 3.82 | 5.87 | 5.27 | 6.32 | 6.52 |
| **Mean change** | -18.83 | -23.58 | -12.43 | -16.43 | -7.97 | -0.78 |
| **95% CI** | -20.60, -17.06 | -25.0, -21.55 | -14.08, -10.78 | -18.74, -14.12 | -9.89, -6.06 | -2.86, 1.29 |
| **SD** | 9.75 | 6.32 | 10.22 | 10.31 | 8.10 | 5.76 |
| **Median** | -21.0 | -24.5 | -11.0 | -19.0 | -7.0 | -0.5 |
| **SRM** | -1.93 | -3.73 | -1.22 | -1.59 | -1.01 | -0.14 |
| **SES** | -3.91 | -6.17 | -2.13 | -3.12 | -1.30 | -0.12 |
| **MADRS as anchor ^a^ (severity criteria #3)** | | | | | | |
| **n** | 113 | 74 | 116 | 39 | 77 | 32 |
| **Mean Baseline** | 26.73 | 27.99 | 26.07 | 24.36 | 26.94 | 26.66 |
| **95% CI** | 25.65, 27.82 | 27.04, 28.93 | 24.94, 27.20 | 21.88, 26.84 | 25.79, 28.08 | 24.31, 29.01 |
| **SD** | 5.81 | 4.09 | 6.13 | 7.66 | 5.03 | 6.52 |
| **Mean change** | -19.43 | -22.15 | -10.07 | -14.28 | -7.94 | -0.78 |
| **95% CI** | -21.18, -17.68 | -23.93, -20.37 | -11.80, -8.34 | -17.60, -10.96 | -9.80, -6.07 | -2.86, 1.29 |
| **SD** | 9.39 | 7.67 | 9.40 | 10.24 | 8.21 | 5.76 |
| **Median** | -21.0 | -24.0 | -9.0 | -15.0 | -7.0 | -0.5 |
| **SRM** | -2.06 | -2.89 | -1.08 | -1.39 | -0.99 | -0.14 |
| **SES** | -3.33 | -5.42 | -1.66 | -1.85 | -1.61 | -0.12 |

^a^ MADRS is grouped as no depression (scores 0 to 12), slight depression (scores 13 to 21), moderate depression (scores 22 to 28) and severe depression (scores >28 to 60); ^b^ MADRS is grouped no depression (scores 0 to 6), mild (scores 7 to 19), moderate (scores 20 to 34) and severe (scores >34 to 60); ^c^ MADRS is grouped as no depression (scores 0 to 12), mild (scores 13 to 17), moderate (scores 18 to 34) and severe (scores >34 to 60). *CAT* category, *CI* confidence interval, *IMP* improvement, *MADRS* Montgomery–Åsberg Depression Rating Scale, *SD* standard deviation, *SRM* standardized response mean, *SES* standardized effect size
